# The splice site variant rs11078928 may be associated with a genotype-dependent alteration in expression of *GSDMB* transcripts

**DOI:** 10.1186/1471-2164-14-627

**Published:** 2013-09-17

**Authors:** Faer S Morrison, Jonathan M Locke, Andrew R Wood, Marcus Tuke, Dorota Pasko, Anna Murray, Tim Frayling, Lorna W Harries

**Affiliations:** 1 RNA mediated mechanisms of disease group, University of Exeter Medical School, EX2 5DW Exeter, UK; 2 Genetics of Complex Traits, University of Exeter Medical School, EX1 2LU Exeter, UK

**Keywords:** GSDMB, Rs11078928, Asthma, Autoimmune disease, GWAS, SNP, Alternative mRNA splicing

## Abstract

**Background:**

Many genetic variants have been associated with susceptibility to complex traits by genome wide association studies (GWAS), but for most, causal genes and mechanisms of action have yet to be elucidated. Using bioinformatics, we identified index and proxy variants associated with autoimmune disease susceptibility, with the potential to affect splicing of candidate genes. PCR and sequence analysis of whole blood RNA samples from population controls was then carried out for the 8 most promising variants to determine the effect of genetic variation on splicing of target genes.

**Results:**

We identified 31 splice site SNPs with the potential to affect splicing, and prioritised 8 to determine the effect of genotype on candidate gene splicing. We identified that variants rs11078928 and rs2014886 were associated with altered splicing of the *GSDMB* and *TSFM* genes respectively. rs11078928, present in the asthma and autoimmune disease susceptibility locus on chromosome 17q12-21, was associated with the production of a novel Δ exon5-8 transcript of the *GSDMB* gene, and a separate decrease in the percentage of transcripts with inclusion of exon 6, whereas the multiple sclerosis susceptibility variant rs2014886, was associated with an alternative *TFSM* transcript encompassing a short cryptic exon within intron 2.

**Conclusions:**

Our findings demonstrate the utility of a bioinformatic approach in identification and prioritisation of genetic variants effecting splicing of their host genes, and suggest that rs11078928 and rs2014886 may affect the splicing of the *GSDMB* and *TSFM* genes respectively.

## Background

Genome wide association studies (GWAS) have greatly increased our understanding of the genetic basis of many complex diseases and traits by identifying single nucleotide polymorphisms (SNPs) that act as susceptibility factors for these diseases [1,2]. However, the results of GWAS only give us a genomic region associated with a particular disease or trait, and the causal SNP, gene and importantly, mechanism of action remain elusive. To date, only a handful of SNPs have been demonstrated as causal for a particular disease and the mechanism whereby the SNP confers disease risk identified. For example, GWAS has identified hundreds of loci associated with susceptibility to inflammatory or autoimmune diseases such as Crohn’s disease, asthma, multiple sclerosis (MS) and type 1 diabetes (T1D), furthering our understanding of the genetic basis of these diseases and identifying areas of the genome for further study [3]. However, apart from a variant in the immunity-related GTPase family, M (*IRGM*) gene, which has been shown to confer susceptibility to Crohn’s disease by altering miRNA binding at the site of the SNP [4], the causal genes and mechanisms in most cases remain to be identified. The slow rate of progress in converting advances made by GWAS into a fuller understanding of which genes are associated with disease, and their mechanistic actions, could be a reflection of the focus on protein coding variants, whereas most SNPs are in fact within non-coding regions of the gene [5,6]. At least 80% of the human genome has now been demonstrated to contain important regulatory sequences such as enhancers, silencers, small RNA binding sites and chromatin modifiers [7-12]. An important mechanism of gene regulation, alternative splicing, is a complex process requiring over 100 splicing factors that recognise regulatory elements such as exonic and intronic splicing enhancers and silencers (ESEs, ESSs, ISEs, ISSs), which bind *trans*-acting splicing regulatory factors [13] that bind to the mRNA secondary structure [14]. Regulatory elements include the splice acceptor AG, the polypyrimidine tract, the splice donor GT and the branch site adenosine residue [13]. In addition disruption of any of these sequences has the potential to alter splicing by re-directing the spliceosome or by altering binding of auxiliary factors to exonic and intronic splicing enhancers and silencers.

In this study, our aim was to follow a bioinformatic pipeline in order to identify autoimmune disease-associated variants with the potential to effect splicing, and to follow up interesting candidate variants by mRNA analysis in whole blood, an appropriate tissue for the analysis of autoimmune or inflammatory disorders. In this article, we report two variants in two genes that exhibit a genotype-associated effect on mRNA processing of their host genes.

## Results

### SNPs associated with autoimmune or inflammatory diseases are enriched for splice site variants

**Figure 1:**
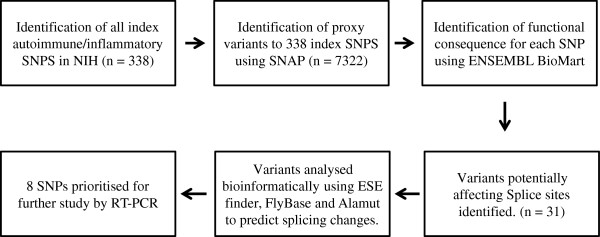
**Bioinformatic pipeline used to predict splice site SNPs that are associated with autoimmune diseases and inflammatory traits.** Index inflammatory SNPs were identified that had been associated by GWAS with susceptibility to autoimmune diseases and inflammatory traits. The proxies to these GWAS SNPs were pulled using the SNAP Proxy Search tool (Broad Institute). The ‘functional consequence to transcript’ for each SNP was identified as being ‘Splice Site’ or ‘Essential Splice Site’ using the Biomart function of Ensembl. These SNPs were then bioinformatically analysed to predict whether a splicing change was likely to occur, using the programmes ESE finder (web-based tool that predicts ESE element sequences that are bound by SR proteins), NNSplice (web-based tool that algorithmically predicts core spice site sequences in a given sequence) and Alamut (splicing mutation prediction programme that amalgamates predictions from five different splice site prediction algorithms to identify potential core splice sites in a given gene transcript). A subset of SNPs was then prioritised for further analysis of their splicing by RT-PCR. The numbers in brackets denote how many SNPs were identified at each stage.

**Figure 2:**
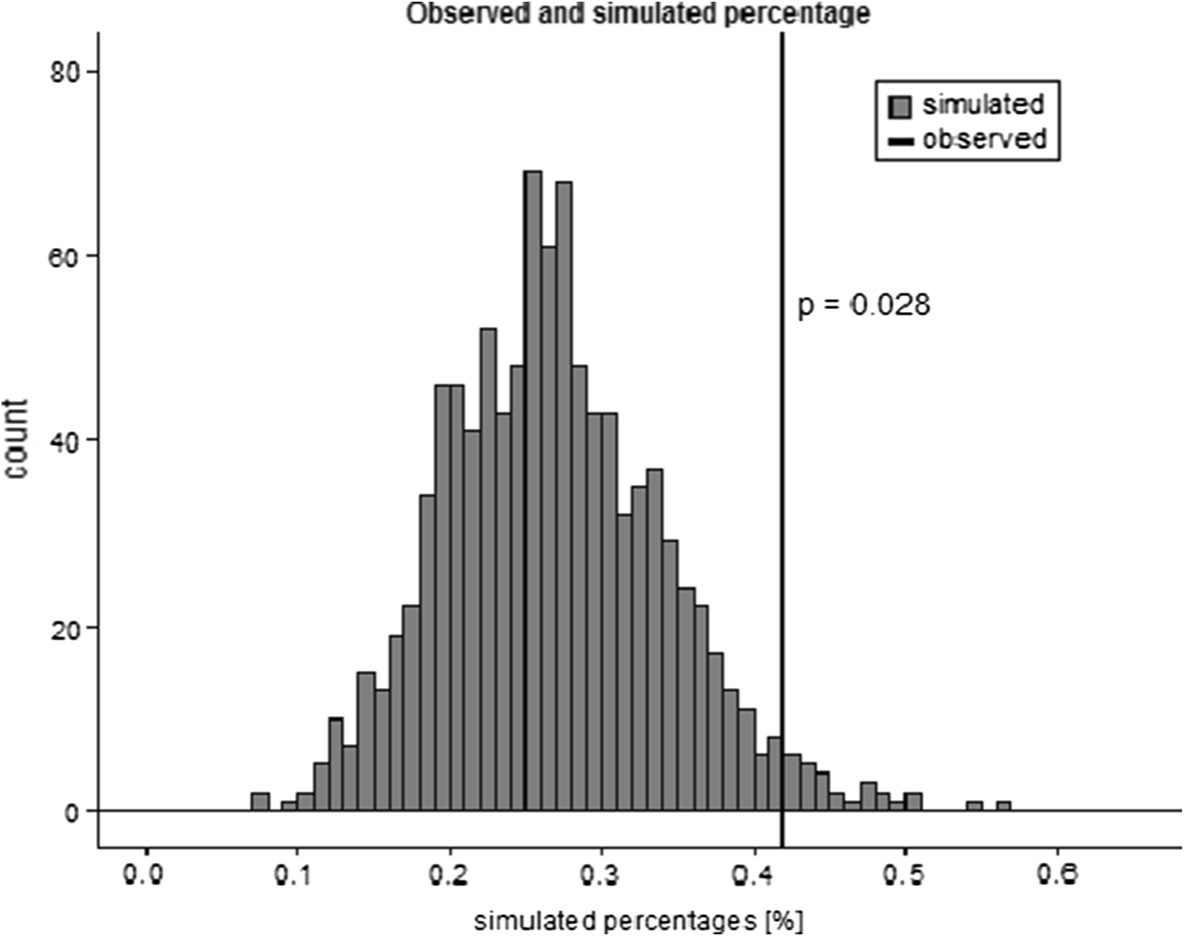
**Enrichment of variants associated with inflammatory or autoimmune phenotypes in splicing control regions.** We assessed the number of randomly selected variants located in splicing control elements by chance. For each set of 338 ‘index’ SNPs and their proxies, the percentage of variants located in ‘splice region elements’ is given on the X-axis (simulated percentage), and the number of SNPs at each percentage (count) is given on the Y-axis. The observed number of autoimmune or inflammatory SNPs located in ‘splice region elements’ is given by the line.

We performed a series of bioinformatic analyses to identify genetic variants that could potentially alter splicing of their host genes (Figure 1). 338 SNPs associated with one or more autoimmune diseases or inflammatory traits were identified that reached genome wide significance, and 7322 proxies were identified to these index SNPs. Of these, 31 SNPs were annotated as *‘splice_region_variant’* using the Biomart program (Ensembl; http://www.ensembl.org). When compared to 1000 sets of 338 random SNPs and their proxies, we found that 0.41% variants associated with autoimmune or inflammatory phenotypes were located in splice regions, compared with a mean of 0.28% for randomly selected variants (one tailed t-test p = 0.028; Figure 2), indicating that inflammatory or autoimmune SNPS were enriched for splice site variants.

### Two SNPs in splice site regions were shown to produce alternative splice products

8 variants were prioritised for further analysis on the basis that they had the potential to create cryptic splice sites, interrupt polypyrimidine tract sequences or disrupt regulatory elements involved in splicing. The 8 variants prioritised for further analysis using this pipeline are shown in Table 1. Unusual bands were identified for half of the variants tested upon RT-PCR (rs11078928 (*GSDMB*), rs2014886 (*TSFM*), rs1260326 (*GCKR*) and rs3764021 (*CLEC2D*). Two bands (*GCKR*, *CLEC2D*) were artifactual, and the remaining two (*GSDMB, TSFM*) were further characterized and sequenced. We found no evidence of altered splicing caused by the remaining variants, although we cannot rule out splicing changes not identified because of primer placement or transcript levels.

**Table 1:** The 8 splice site SNPs prioritised for further analysis after following the bioinformatic pipeline set out

**Proxy (i.e. splice site SNP)**	**GWAS variants in LD with splice site SNPs**	**P value**	**Phenotype (risk allele)**	**PID**	***r***^**2**^	**Host gene of splice site SNP**	**Alleles for splice site SNP**	**Consequence to transcript**	**Prediction/location**
rs11078928	rs2872507	5 × 10^-11^	Ulcerative colitis (A)	21297633	1	GSDMB	A > G	Essential Splice Site	Destroys AG of acceptor splice site intron 5: Predicted skip of exon 6 (Alamut)
	rs2872507	5 × 10^-9^	Crohn’s disease (A)	18587394	1				
	rs9303277	2 × 10^-9^	Primary biliary cirrhosis (T)	20639880	0.905				
	rs2290400	6 × 10^-13^	Type 1 diabetes (?)	19430480	0.905				
	rs7216389	9 × 10^-11^	(Asthma (T))	17611496	0.905				
rs2014886	rs703842	5 × 10^-11^	multiple sclerosis (A)	19525955	0.964	TSFM	C > T	Essential Splice Site	Introduces cryptic donor splice site intron 2: Likely to result in alternative exon inclusion
rs1260326	rs780094	7 × 10^-15^	C-reactive protein (A)	18439548	0.933	GCKR	T > C	Splice Site	1 bp from GT of splice donor exon 15, alters splice site score
	rs780093	5 × 10^-11^	Crohn’s disease (T)	21102463	0.901				
rs10263341	rs886774	3 × 10^-8^	Ulcerative colitis (G)	19915572	0.803	DLD	T > C	Splice Site	May interrupt polpyrimidine tract intron 6
rs1322077	rs2301436	1 × 10^-12^	Crohn’s disease (T)	18587394	0.904	FGFR1OP	T > C	Splice Site	May interrupt polpyrimidine tract intron 5
rs2020854	rs2066808	1 × 10^-9^	Psoriasis (A)	19169254	1	STAT2	A > G	Splice Site	May introduce cryptic donor site intron 14
rs55719896	rs8049439	2 × 10^-9^	Inflammatory bowel disease, early onset (G)	19915574	0.965	ATXN2L	G > A	Splice Site	Destroys AG of cryptic acceptor splice site intron 20
	rs4788084	3 × 10^-13^	Type 1 diabetes (G)	19430480	0.824				
rs3764021	rs3764021	5 × 10^-8^	Type 1 diabetes (G)	17554300	1	CLEC2D	C > T	Splice Site	May introduce cryptic donor splice site exon 2

The table shows the 8 SNPs that lie within splice sites of the genes listed. The index GWAS autoimmune/ inflammatory SNPs that are in LD (*r*^2^ < 0.8) with the splice site SNPs are shown, as well as the splice site prediction/location. *PID* PubMed Accession, *LD* linkage disequilibrium.

### Rs11078928 causes two separate splicing changes in the Gasdermin B (GSDMB) gene

**Figure 3:**
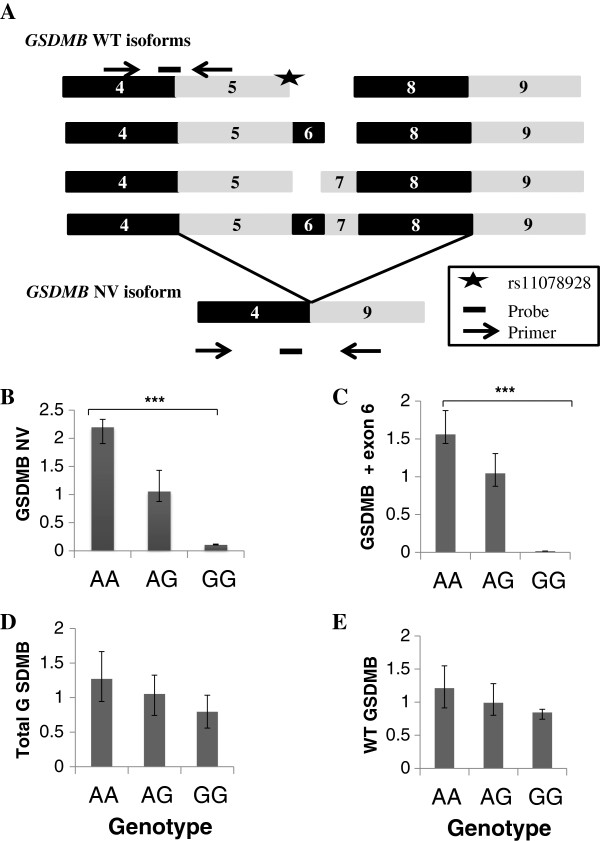
**Wild-type (WT) and Novel Variant (NV) isoforms of the *GSDMB *gene and their expression with genotype. A**. Showing exons 4–9 of the four RefSeq isoforms and the novel *GSDMB* transcipt (*GSDMB* NV), which is missing exons 5–8. Half the Reference Sequence (WT) transcripts include exon 6 and two are lacking exon 6; NM001165958.1 is the full length transcript. The star shows the position of rs11078928 (acceptor splice site of intron 5). The location of the Custom Taqman Assay Primers (indicated by an arrow) and probe (indicated by a rectangle) are shown. **B**. Chart showing the expression of the novel transcript *GSDMB* NV by genotype. Homozygotes for the minor allele show negligible expression of the novel transcript. Expression is normalised to the endogenous control *RPLPO,* and is shown relative to the expression of *GSDMB* NV in heterozygotes. Significant results (P < 0.05) are indicated by an asterix. **C**. Chart showing the expression with genotype of the *GSDMB* transcripts which include exon 6 (NM001165958.1, NM001165959.1). Homozygotes for the minor allele show no expression of exon 6-containing transcripts. Expression is normalised to the endogenous control *RPLPO*, and is shown relative to the expression of exon 6 *GSDMB* transcripts in heterozygotes. **D-E**. Charts showing total expression of *GSDMB* (all RefSeq isoforms, including novel) and expression of *GSDMB* WT (all RefSeq isoforms). Expression is normalized to the endogenous control *RPLPO*, and is shown relative to total and WT expression respectively in heterozygotes. Both show a decrease in expression with the minor allele, although these results did not reach statistical significance.

We found two separate splicing changes associated with the variant rs11078928 in the gene *GSDMB*. These changes include production of a novel transcript of *GSDMB* and a change in isoform ratio. The novel band associated with rs11078928 was found upon sequence characterisation to be a large deletion product lacking exons 5–8 of the gene *GSDMB* (Figure 3A and Additional file 1). To determine whether this deletion was associated with genotype and therefore caused by the variant rs11078928, we designed Taqman assays to the wild-type and novel transcripts as described above from the custom assay service available from Life Technologies (Life Technologies, Foster City, USA). We found evidence to suggest a genotype-associated effect on the expression levels of this deletion product; with homozygotes for the major (A) allele, showing increased expression of this alternate Δ5-8 transcript, and homozygotes for the minor (G) allele showing negligible expression of the transcript compared with heterozygous individuals or those homozygous for the A allele (p = 0.0001; Figure 3B).

Moreover, four reference transcript sequences are described for this gene in expressed sequence tag (EST) databases; two transcripts (NM_001165958.1 and NM_001165959.1) include exon 6, whilst this exon is deleted in the remaining two (NM_001042471.1 and NM_018530.2). Therefore, we quantified relative expression of these different isoforms according to genotype using Taqman Assays (Life Technologies, Foster City, USA) (Additional file 2). Although there was no significant change in expression of the isoforms lacking exon 6, we found that the isoforms which include exon 6 have almost no expression in homozygotes for the minor allele of rs11078928 compared with heterozygous individuals, and those homozygous for the A allele (p = 0.0002; Figure 3C). This striking genotype-specific expression difference suggests that rs11078928 or an associated variant may be altering exon 6 inclusion in the *GSDMB* transcript. The Alamut Mutation Interpretation Software (Interactive Biosoftware, Rouen, France) predicted that rs11078928 would result in deletion of exon 6. We also quantified expression of the WT transcript and overall expression of *GSDMB*, to determine whether these were altered by genotype. The expression differences between genotypes showed decreased expression for those individuals carrying the minor allele, although they did not reach statistical significance (results shown in Figure 3D-E).

Rs2014886 creates a cryptic donor splice site and alters splicing of the Ts translation elongation factor, mitochondrial (TSFM) gene.

**Figure 4:**
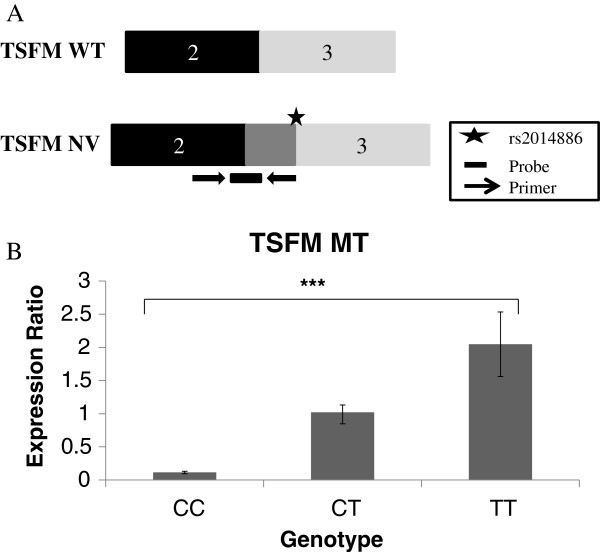
**WT and NV isoforms of *TSFM *and their expression with genotype. A**. Showing exons 2–3 of the RefSeq transcript (there are four RefSeq isoforms for *TSFM*, however, they are identical over this region of the gene) and the novel *TSFM* transcript (*TSFM* NV), which has an alternate 38 bp exon included between introns 2 and 3. The star shows the relative position of rs2014886 (introducing an intronic donor splice site within intron 2. **B**. Chart showing the expression of the novel transcript *TSFM* NV by genotype. Homozygotes for the major allele show negligible expression of the novel transcript. Expression is normalised to the endogenous control *RPLPO,* and is shown relative to the expression of *TSFM* NV in heterozygotes. Statistical significance is indicated by an asterix.

We identified a novel band in carriers of the ‘T’ allele for rs2014886 which we were unable to isolate by conventional means due to the large size of the product. The size of the band (~530 bp) corresponded to a *TSFM* transcript with the inclusion of a short exon, where the donor site is created by rs2014886 and which has EST (expressed sequence tag) evidence of use [15], but which is not present in the reference sequence (RefSeq) transcripts. This donor site is preceded by a strong acceptor site that is present 38 bp upstream of the variant, which also has evidence of use in EST databases [15] (Figure 4A and Additional file 3). We therefore designed RT-PCR primers specific to the predicted novel transcript, to confirm its identity. By this method, we were able to sequence the product and verify that the transcript includes a short 38 bp exon insertion within intron 2 of *TSFM* (Figure 4A). Expression of the novel transcript was found to be associated with genotype, with individuals carrying two minor (T) alleles expressing the novel transcript at levels approximately 2-fold higher than individuals carrying two major (C) alleles (p = 0.00005; Figure 4B). However, we found no significant differences in expression between individuals of different genotypes for expression of total *TSFM* and *TSFM* WT.

## Discussion

Although hundreds of SNPs have been associated with autoimmune diseases by GWAS [1,2] the causal gene and mechanism in most cases remains elusive. We prioritised 8 variants with bioinformatic evidence to alter splicing by creating cryptic splice sites, interrupting polypyrimidine tract sequences or disrupting regulatory elements involved in splicing. Here we provide evidence that a bioinformatic pipeline may be followed in order to identify and prioritise variants that are likely to affect splicing of their host gene. We have identified a genotype-associated effect on alternative splicing for two of the 8 SNPs (rs11078928 and rs2014886) on the processing of the *GSDMB* and *TSFM* genes respectively.

Variant rs11078928 is associated with two separate splicing changes in the gene *GSDMB*, which could possibly be of functional significance, whereas, the effect of rs2014886, within *TSHM* most likely has little functional consequence, since it is not affected by genotype. For the remaining 6 SNPs, we found no evidence for altered splicing. This could indicate that the SNPs in or near these splice sites were too weak to re-direct the splicing machinery, although it cannot be overlooked that the sensitivity of the assays may have affected identification of splice variants (especially at low levels of expression). Alternatively, these genes may be differentially expressed in a tissue other than blood. Nevertheless, variation in splicing regulatory sequences leading to aberrant splicing is a relatively common occurrence and may explain some of the signals identified by GWAS [16].

Variant rs2014886, a C > T change in intron 2 of the *TSFM* gene, was predicted to create a cryptic donor splice site within the intron, and transcripts derived from the use of this cryptic splice site, 38 bp downstream of a strong acceptor splice site, are represented in EST databases [15]. We confirmed that the minor allele of the SNP leads to the production of this alternative splice product, which includes the 38 bp short exon within intron 2. Negligible amounts of the insertion product (referred to as *TSFM* NV) were expressed from the transcripts carrying the major (C) allele (Figure 4B). The difference in age between genotypes in our cohort for the variant rs2014886 reached statistical significance, so it is possible that the effects we note for *TSFM* splicing could be driven by differences in mean age rather than genotype. The insertion of the novel 38 bp intron causes a frameshift event leading to the generation of a premature termination codon at a position 85 bp downstream of the insertion. This would in all probability render the novel transcript susceptible to the nonsense-mediated decay mRNA surveillance pathway [17]. *TSFM* has been identified as a likely candidate gene in multiple sclerosis susceptibility [18], and its expressio14, para 2n was recently found to be correlated with a variant that alters an enhancer region [19]. Therefore, since overall expression of all *TSFM* isoforms and of the four reference sequence isoforms (*TSFM* WT) was not significantly altered by genotype, we conclude that the exon inclusion caused by the SNP is unlikely to be of functional significance.

**Figure 5:**
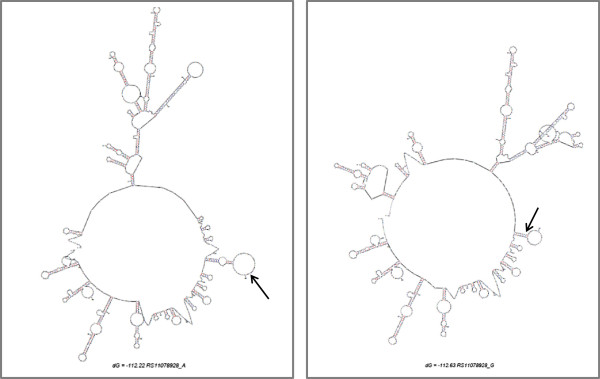
**Showing the secondary structure change with the major A allele (right) and the minor allele G (left), as predicted by Mfold (**http://mfold.rna.albany.edu/?q=mfold**).** The arrows indicate the position of the variant rs11078928 in the *GSDMB* transcript sequence.

The genotype-specific splicing changes of the *GSDMB* gene may be of more potential importance. Variant rs11078928, an A > G change in the acceptor splice site of intron 5 of the *GSDMB* gene, is associated with two separate alternative splicing events. Firstly the major (A) allele of rs11078928 is associated with exon skipping, resulting in a splice product missing exons 5–8 (referred to as *GSDMB* NV), whereas this does not occur in individuals carrying the minor (G) allele. The cause of the deletion is not easy to ascertain, but could be attributable to changes in the RNA secondary structure induced by this SNP, which could alter binding of the splicing machinery (Figure 5). It is also possible that another SNP or SNPs in linkage disequilibrium (LD) with rs11078928 may be causing this splicing alteration. Although WT *GSDMB* and total *GSDMB* expression was decreased in those carrying the minor allele, these changes did not reach statistical significance, indicating that the disease susceptibility may be attributable to a gain of function effect from the novel transcript, rather than the amount of the *GSDMB* transcript pool (Figure 3). It is difficult however to speculate as to the effect of this large deletion on GSDMB protein function, since little is known about the structural function of this protein. Secondly, we identified that subjects carrying the minor (G) allele of rs11078928 exhibit skipping of exon 6 of the gene, with homozygotes for the minor allele expressing very little full length product (transcripts containing exon 6).

Taken together, these changes indicate that whilst individuals homozygous for the major (A) allele do express the Δ5-8 deleted *GSDMB* product, they also express the full length exon 6-containing isoforms. Conversely, individuals homozygous for the minor (G) allele do not express either the deleted *GSDMB* product or the full length product (isoforms containing exon 6), as reflected in their lower overall expression. This may suggest some functional importance of the region of the GSDMB protein encoded by exon 6, but there is very little information on the nature of this region in the current literature.

*GSDMB* encodes for the protein Gasdermin B, which is a member of the gasdermin-domain containing protein family. Although the exact function of GSDMB remains unclear, members of the gasdermin-domain containing family have roles in epithelial cell apoptosis and in maintaining a differentiated state of epithelial cells [20]. GSDMB has also been shown to be important in cancer pathogenesis, with alternative splicing of the gene being involved in gastrointestinal and hepatic cancers [21]. The *GSDMB* gene contains several conserved amino acid sequences in the N and C terminal regions as well as several conserved leucine-rich motifs throughout the sequence [22]. The deletion product described here, *GSDMB* NV, is in frame so would not be subject to nonsense-mediated decay and could plausibly be translated into the protein.

Variant rs11078928 is located on chromosome 17q12-21, in an area which was identified in 2007 to be associated with asthma susceptibility (rs7216389), as well as susceptibility to developing several autoimmune diseases, including ulcerative colitis (rs2872507), Crohn’s disease (rs2872507), type 1 diabetes (rs2290400) and primary biliary cirrhosis (rs9303277) (see Table 1) [23]. It is interesting to note that the risk alleles for allergic disease susceptibility are the alternative to that for autoimmune disease susceptibility, indicating opposite effects of the variants on pathogenesis of these diseases [24]. The minor allele for rs11078928, increases risk for developing these autoimmune traits (risk allele for type 1 diabetes not known) and confers a decreased risk to developing asthma. This association has been replicated several times by independent groups and in different populations, and there has been much debate over potential mechanisms of action and which is the causal gene(s) in that locus.

*GSDMB*, *ORMDL3* (ORM1-like 3) and *ZPBP2* (zona pellucida binding protein 2) have all been considered good candidates, as they are all expression quantitative trait loci, with opposite directions of expression seen with genotype for *ORMDL3*/*GSDMB* and *ZPBP2*. At present, the best functional evidence of the mechanism by which the 17q12-21 locus may alter asthma and autoimmune disease susceptibility comes from two studies by the same group, who identified a 5.3 kb region overlapping the *ZPBP2* gene, which is associated with several gene regulatory marks, including allele-specific chromatin interactions, DNA methylation and promoter activity [20,25]. It has been hypothesised that this regulatory region may be involved in long-range chromatin interactions and may influence the expression of any one or all of the genes *GSDMB*, *ORMDL3* and *ZPBP2*[20,25].

*ZPBP2* has a well-known role in fertilisation and male fertility, and it has been proposed that this gene may play a role in influencing the prevalence of asthma in the population [25]*. ORMDL3* has so far been thought to be the most promising candidate at this locus, since it has a role in mediating inflammation, and also has expression in bronchial epithelial cells, where slightly higher *ORMDL3* expression was found in individuals with asthma compared to controls [26,27]. *GSDMB* shows a diverse expression pattern in tissues, including expression in the lung, liver, intestine and colon, but with very low expression in bronchial epithelial cells. However, it has been suggested that the effect of the chromosome 17q12-21 locus on multiple diseases may reflect a more direct role on immune function rather than any tissue-specific effect [20,28]. The expression of *GSDMB* in the thymus and CD8+ and CD4+ T-cells would increase the plausibility of this inference, especially given the involvement of type 1 and type 2 immune responses in autoimmune and allergic disease respectively [20,29].

Although genome-wide analysis of transcript isoform expression in the CEU HapMap population found no differences in the expression of *GSDMB* isoform ratios associated with the asthma haplotype in LCLs (lymphoblastoid cell lines) [30], the splicing of *GSDMB* has been shown to be associated with genotype of the related variant rs7216389 in brain and in peripheral blood mononucleated cells [31]. rs7216389 is in the same LD block as rs11078928 (*r*^2^ 0.905, D’1.0), and our data thus suggest the effect on splicing noted in this, and in our study may be an effect of the splice site SNP rs11078928. Interestingly Heinzen *et al.* found an overrepresentation of autoimmune traits associated with splicing quantitative trait loci (sQTLs), which could indicate the importance of splicing differences in these diseases [31]. Finally, LCLs may not offer a realistic representation of regulation of *in vivo* gene expression [32], explaining the differences in *GSDMB* splicing patterns seen in primary cell types and LCLs.

These data are of course preliminary in nature, and require further work to assess the potential consequences of a reduction in *GSDMB* expression, or an alteration to the pool of transcripts expressed from the *GSDMB* gene to the function of tissues involved in immune function and inflammation. Future studies should focus on the effect of alterations to the *GSDMB* isoform pool on factors such as the inflammatory milieu in other appropriate cell types to prove causality and further define mechanism. It remains to be seen also if *GSDMB* splicing changes are also noted in individuals with asthma and autoimmune diseases.

## Conclusions

To conclude, we have demonstrated that a proportion of genetic variants identified as susceptibility loci for inflammatory or autoimmune disease may act by disrupting the native splicing patterns of their host genes. Using bioinformatics to identify variants likely to interfere with splicing patterns, followed by functional evaluation in whole blood, we have demonstrated alterations in the splicing of the *GSDMB* and *TSFM* genes, which is associated with genotype at rs11078928 and rs2014886 respectively. Although the exon inclusion caused by the variant in *TSFM* is unlikely to have any functional significance, our data suggest that rs11078928 is associated with the production of a novel *GSDMB* transcript lacking an internal segment, together with a change in the ratio of some known isoforms. This is predicted to result in an almost complete lack of full length *GSDMB* mRNA in individuals homozygous for the minor allele. Although the functional significance of these changes remains to be determined, our study provides further evidence that *GSDMB* is a promising candidate gene at the 17q12-21 locus, in altering susceptibility to various autoimmune diseases, and asthma.

## Methods

### Identifying candidate genes for splicing analysis

We searched the National Institutes of Health (NIH) GWAS catalogue (http://www.genome.gov/gwastudies) [5] to identify SNPs associated with known autoimmune diseases at genome wide significance (*P* < 5×10^-8^). We focussed on inflammatory and autoimmune traits as we had access to an appropriate tissue, i.e. lymphocyte RNA, to assess splicing. We defined autoimmune diseases/ inflammatory traits as those described in the literature as ‘autoimmune disease’ or ‘chronic inflammatory disease’. Next we identified proxy SNPs to these candidates that had *r*^2^ >0.8, and located within 500 kb of the index variant, using the SNAP Proxy Search tool (http://www.broadinstitute.org/mpg/snap/ldsearch.php) [33]. The functional consequence of each SNP on transcription was then identified using the BioMart function of Ensembl (http://www.ensembl.org) (Homo sapiens Variation (dbSNP build 135; ENSEMBL). SNPs labelled as ‘Splice Site’ or ‘Essential Splice Site’ were identified and these SNPs were analysed *in silico* using ESE Finder [34,35], Alamut Mutation Interpretation Software (Interactive Biosoftware, Rouen, France), and Flybase from the Berkeley Drosophila Genome Project (http://www.fruitfly.org/seq_tools/splice.html) [36], in order to predict how likely a splicing change would occur as a result of the SNP (Figure 1 and Table 1). Variants demonstrating good evidence of the capacity to modify splicing patterns were prioritised for further transcriptomic analysis in mRNA derived from whole blood samples from subjects of defined genotype.

### Assessment of splice site enrichment for SNPs associated with inflammatory or autoimmune diseases

To determine the likelihood that SNPs associated with autoimmune or inflammatory phenotypes were located in elements responsible for splice site choice by chance alone, we selected 1000 sets of 338 random SNPs matched on allele frequency (± 5%) and gene proximity (± 10 kb). For each set we retrieved all variants in linkage disequilibrium (r^2^ >0.8 in 1000 Genomes Phase 1 data in Europeans) and used them to query the BioMart database using the *‘biomaRt’* package. In each set we calculated the proportion of SNPs identified by BioMart as *‘splice_region_variant’*. Finally, we compared the proportion of splice site variants in 338 autoimmune disease SNPs with proportions identified in 1000 sets of matched proxies and observed enrichment for splice site variants by one tailed t-test.

### Cohort information and sample collection

The samples used were from the Exeter 10,000 cohort (http://www.exeter.crf.nihr.ac.uk/). *GSDMB* and *TSFM* total expression, expression of wild-type (WT) and novel (NV) transcripts, as well as *GSDMB* isoform specific expression, were compared between 8–10 individuals homozygous for the major alleles, 8–10 heterozygous individuals, and 5–8 individuals homozygous for the minor alleles. The individuals included in the cohort were of mixed gender and were of predominantly White British origin. 20% of the *GSDMB* WT, NV and isoform-specific expression cohort had diagnosed autoimmune disease (AA n = 2, AG n = 2 and GG n = 2), as had 23% of the *GSDMB* total expression cohort (AA n = 3, AG n = 2 and GG n = 0) and 21% of the TSHM cohort (CC n = 3, CT n = 1 and TT n = 1). The median ages were as follows: *GSDMB* WT, NV and isoform-specific expression: 62 (AA n = 10) 61 (AG n = 10) 60 (GG n = 5); *GSDMB* total expression (expression of WT and alternative transcripts) 60 (AA n = 8) 56 (AG n = 8) 54 (GG n = 6); *TSFM* WT, NV and total expression 46 (CC n = 8) 63 (CT n = 8) 37 (TT n = 8). The median ages between genotypes were compared using the Kruskal-Wallis H test and did not reach statistical significance (P < 0.05) in the case of *GSDMB*. However, when comparing the median ages for the *TSFM* cohort, the differences between the groups of each genotype did reach statistical significance (P = 0.04). The median BMIs were as follows: *GSDMB* WT, NV, isoform-specific expression: 27 (AA) 25 (AG) 26 (GG); *GSDMB* total expression 25 (AA) 26 (AG) 25 (GG); *TSFM* WT, NV and total expression 25 (CC) 29 (CT) 22 (TT). The median BMIs were compared using the Kruskal-Wallis H test and did not reach statistical significant for any of the groups. Median white blood cell counts were compared using Kruskal-Wallis H test and did not reach statistical significance across any of the groups. Data and statistics for these parameters are given in Additional file 4. Current medication and past medical history were known for the donors. 2.5 ml peripheral blood specimens were collected using PAXgene technology [37] and extracted using the PAXgene Blood mRNA kit (Qiagen, Crawley, UK) according to the manufacturer’s instructions. Research was carried out in accordance with the Helsinki Declaration, and ethical approval was granted by the Bristol Regional Ethics Committee (study number 09/H0106/75). Written informed consent was obtained for all participants.

### SNP genotyping

PCR primers were designed to the area surrounding the SNP. The primer sequences were as follows: *GSDMB* forward (5′-GGTGCGTCTTACCACATCCT-3′), *GSDMB* reverse (5′-GGGACTGGAGAAAGGGAACT-3′); *TSFM* forward (5′-GGCGAAACCCCATCTCTACT-3′), *TSFM* reverse (5′- CCCCCACACTGTCTGACTTT-3′); *GCKR* forward (5′- CCCTCCCCTTCTCCTAGACA-3′), *GCKR* reverse (5′- GCTGATGATGGAGGGAAAGA-3′); *DLD* forward (5′- CCTGAAATAGATTTCCCTGACA-3′), *DLD* reverse (5′- GCCATCAGCTTTCGTAGCAG-3′); *FGFR1OP* forward (5′- GAAGGTTTTTGAGGGGGTAAA-3′), *FGFR1OP* reverse (5′- TTTCCCTCTGGTGACTTTGG-3′); *STAT2* forward (5′- CACAGACTCTGGTGGAGCAA-3′), *STAT2* reverse (5′- TGCAGTTCCTCTGTCACACC-3′); *ATXN2L* forward (5′- TGGCCAGAAGAAGGGATAGA-3′), *ATXN2L* reverse (5′-AGATTCTGCTGTGGCTGTCC-3′); *CLEC2D* forward (5′-GGTGCCACTTAAAAAGTTATTGG-3′), *CLEC2D* reverse (5′- AGTGTGTGGAATGGTTGCTG-3′). 50 ng DNA from the peripheral blood of healthy controls was amplified by PCR using MegaMix Royal (Cambio, Cambridge, UK) under the following conditions: 95°C for 5 min; followed by 40 cycles of: 95°C for 30s, 60°C for 1 min, 72°C for 1 min; followed by 72°C for 10 min. PCR products were then sequenced to confirm genotype. Sequence analysis was carried out using the Mutation Surveyor software package (SoftGenetics, Pennsylvania, USA).

### RT-PCR and band isolation to identify novel splice products

PCR primers were designed to at least 2 exons either side of the SNP of interest. The primer sequences were as follows: *GSDMB* forward (5′- ACCCTTTTCATTCCGATCAA-3′), *GSDMB* reverse (5′- AAGTCCAGAATGGCTTTTGC-3′); *TSFM* forward (5′- GGTGTTTATCGCGGCTAGAG -3′), *TSFM* reverse (5′- CAGAGGGTTGATCCTTTAGGG -3′); *TSFM* NV forward (5′- GACCTCAAACAGACGGAGTCTTGCT-3′), *TSFM* NV reverse (5′- TCTTTGGTCTTCCTCCCTTGGAGC-3′), *GCKR* forward (5′- GGCTTTCTCATTGGTGATCACAGTGA-3′), *GCKR* reverse (5′- AGCTTGGAGTTGCTAATCCGAAGGT-3′); *DLD* forward (5′- CTTGGTGGAACATGCTTGAA-3′), *DLD* reverse (5′- CCATCTGACTTCTTGGTAGCAC-3′), *FGFR1OP* forward (5′- TCCTTTAGTTAATGAGAGCCTGAAA-3′), *FGFR1OP* reverse (5′ CAGACTTCCTGCTTGCTTCC-3′); *STAT2* forward (5′- TCCTCCTCAATTACAAGGCTTC-3′), *STAT2* reverse (5′- TGCTCAGCTGGTCTGAGTTG-3′); *ATXN2L* forward (5′- CCAAGCCCTTTATGCCACT-3′), *ATXN2L* reverse (5′- GAAGCTGCTCTGAGGGGACT-3′); *CLEC2D* forward (5′- AACCCAGGTTGTCTGCATTC-3′), *CLEC2D* reverse (5′- TTCAGTACCATTTATCCATTTCCA-3′). 100 ng peripheral blood RNA from individuals of known genotype, was reverse transcribed using SuperScript®III First-Strand Synthesis System following the protocol as recommended by the manufacturer (Invitrogen by Life Technologies, Foster City, USA). The cDNA was then amplified by PCR using MegaMix Royal (Cambio, Cambridge, UK) under the following conditions: 95°C for 5 min; followed by 30 cycles of: 95°C for 30 s, 60°C for 1 min, 72°C for 1 min; followed by 72°C for 10 min, and the products were separated on a 2% agarose gel. Novel bands were identified, isolated and amplified using the respective PCR primers and conditions already described. The amplification products were then sequenced to confirm identity and characterise any unusual products.

### Real-time PCR to quantify relative expression of isoforms by genotype

Real-time PCR primers were designed to the reference sequence (wild-type; WT) and any novel (novel; NV) transcripts. Where alternatively expressed isoforms were present in the region of interest, inventoried Taqman Assays were purchased (Life Technologies, Foster City, USA), information for which is shown in Additional file 2. Assays for total expression of all wild-type transcripts as well as the novel transcripts were also purchased. Primer and probe information for Custom Taqman Assays are given in Additional file 5, and their relative positions are indicated in Figures 2 and 3. RNA was reverse transcribed as described previously, and real-time quantitative PCR was performed using the ABI Prism 7900HT platform. RNA from 5–10 individuals of each genotype was amplified by this method (10X heterozygotes, 10X homozygotes for the major allele, 5-10X homozygotes for the minor allele). Results were analysed using the Comparative Ct method [38] to identify genotype-specific alternations in transcript levels.

### Assessment of secondary structure change

Pre-spliced hnRNA secondary structure was analysed using MFOLD software (http://www.bioinfo.rpi.edu/applications/mfold/), under default settings [39,40].

### Statistical analysis

Differences between the expression patterns of normal and aberrant transcripts according to genotype were assessed by the Kruskal-Wallis H test. Non-parametric statistics were necessary due to the relatively small sample numbers and the expected non-normality of data.

## Abbreviations

SNP: Single nucleotide polymorphism; GWAS: Genome Wide Association Studies; LD: Linkage disequilibrium; GSDMB: Gasdermin B; TSFM: Ts translation elongation factor, mitochondrial; WT: Wild-type; MT: Mutant; NV: Novel variant; LCL: Lymphoblastoid cell line

## Competing interests

The authors declare that they have no competing interests.

## Authors’ contributions

FSM carried out all experimental and statistical analyses, and was responsible for drafting the manuscript. JML participated in design of the study, and participated in its coordination and implementation. ARW and MT participated in the bioinformatic analyses. DP carried out the assessment of splice site enrichment for SNPs associated with inflammatory and autoimmune diseases. AM and TF contributed to the study, and participated in its design and coordination. LWH managed the study, oversaw final approval of the manuscript and contributed funding. All authors read and approved the final manuscript.

## Supplementary Material

Additional file 1 **(Additional data file 1.ppt) is a figure showing the difference in nucleotide sequence between *GSDMB* WT and *GSDMB *NV transcript sequences.** The title of the figure is “Electropherogram showing changes to the *GSDMB* transcript at the sequence level”. Click here for file

Additional file 2 **(Additional data file 2.csv) is a table showing the Taqman Assay information, including Assay IDs (Applied Biosystems, Foster City, USA) along with information about the transcript(s) that the assay will detect.** The title of the table is “Taqman Assay information”. Click here for file

Additional file 3 **(Additional data file 3.ppt) is a figure showing the difference in nucleotide sequence between *TSFM *WT and *TSFM *NV transcript sequences.** The title of the figure is “Electropherogram showing the changes to the *TSFM* transcript at the sequence level”. Click here for file

Additional file 4 (Additional data file 4.docx) is a table giving the age, BMI, white blood count, Platelet count and haemoglobin measurements for study participants. Click here for file

Additional file 5 **(Additional data file 5.csv) is a table listing the Custom Taqman Assay information, including primer and reporter information, and transcripts detected by the Custom Taqman Assays, provided by Applied Biosystems (Foster City, USA).** The title of the table is “Custom Taqman Assay information for *GSDMB* and *TSFM*”. Click here for file
