# Supplementary figures and images for: The splice site variant rs11078928 may be associated with a genotype-dependent alteration in expression of GSDMB transcripts
Source: BMC Genomics. 2013 Sep 17;14:627. doi: 10.1186/1471-2164-14-627 (PMC3848490; doi:10.1186/1471-2164-14-627)

## Slide 1
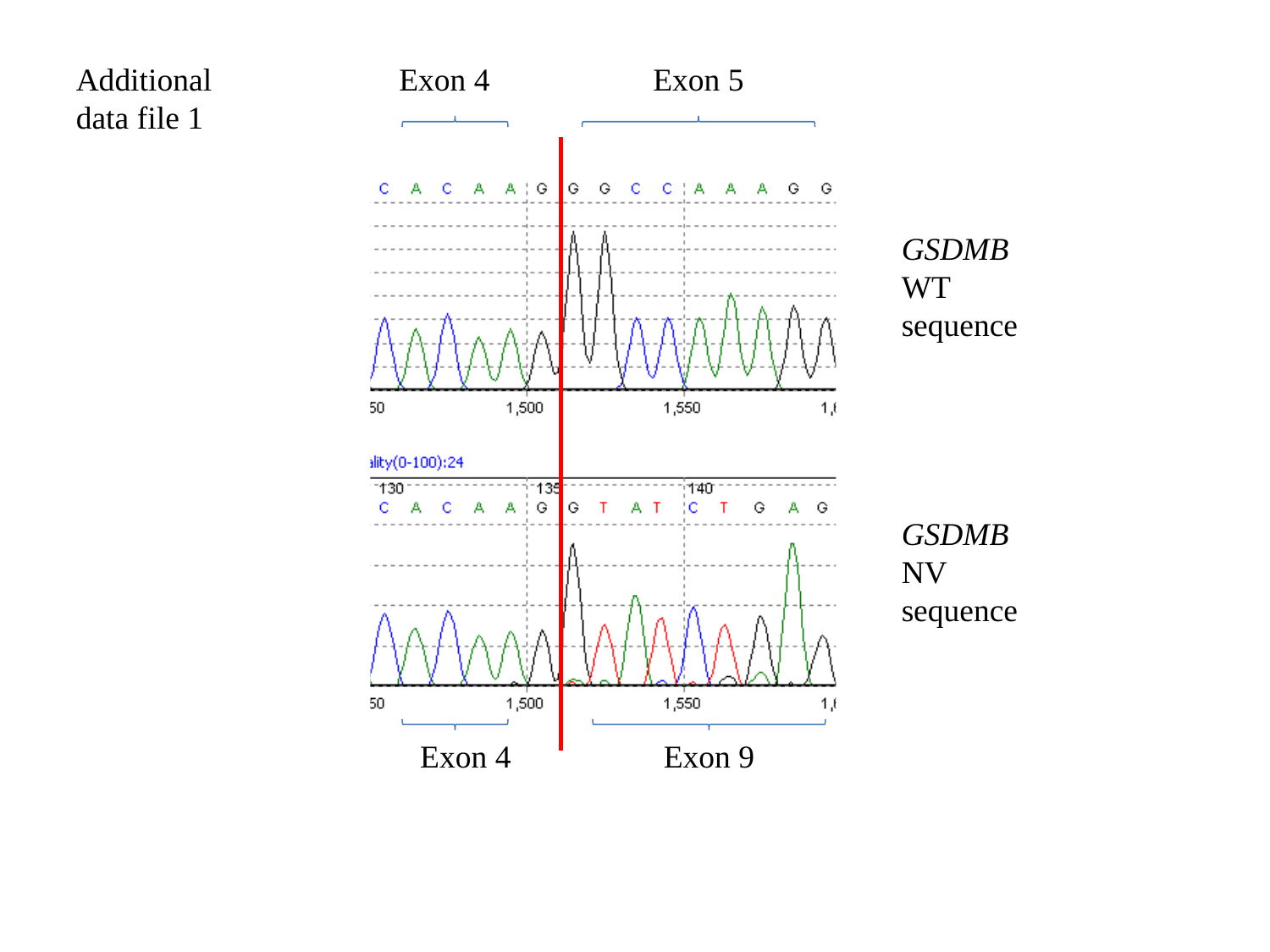

Additional data file 1
Exon 4
Exon 5
GSDMB WT sequence
GSDMB NV sequence
Exon 4
Exon 9

Supplement: Additional file 1 — (Additional data file 1.ppt) is a figure showing the difference in nucleotide sequence between GSDMB WT and GSDMB NV transcript sequences. The title of the figure is “Electropherogram showing changes to the GSDMB transcript at the sequence level”. [file 1471-2164-14-627-S1.pptx]

## Slide 1
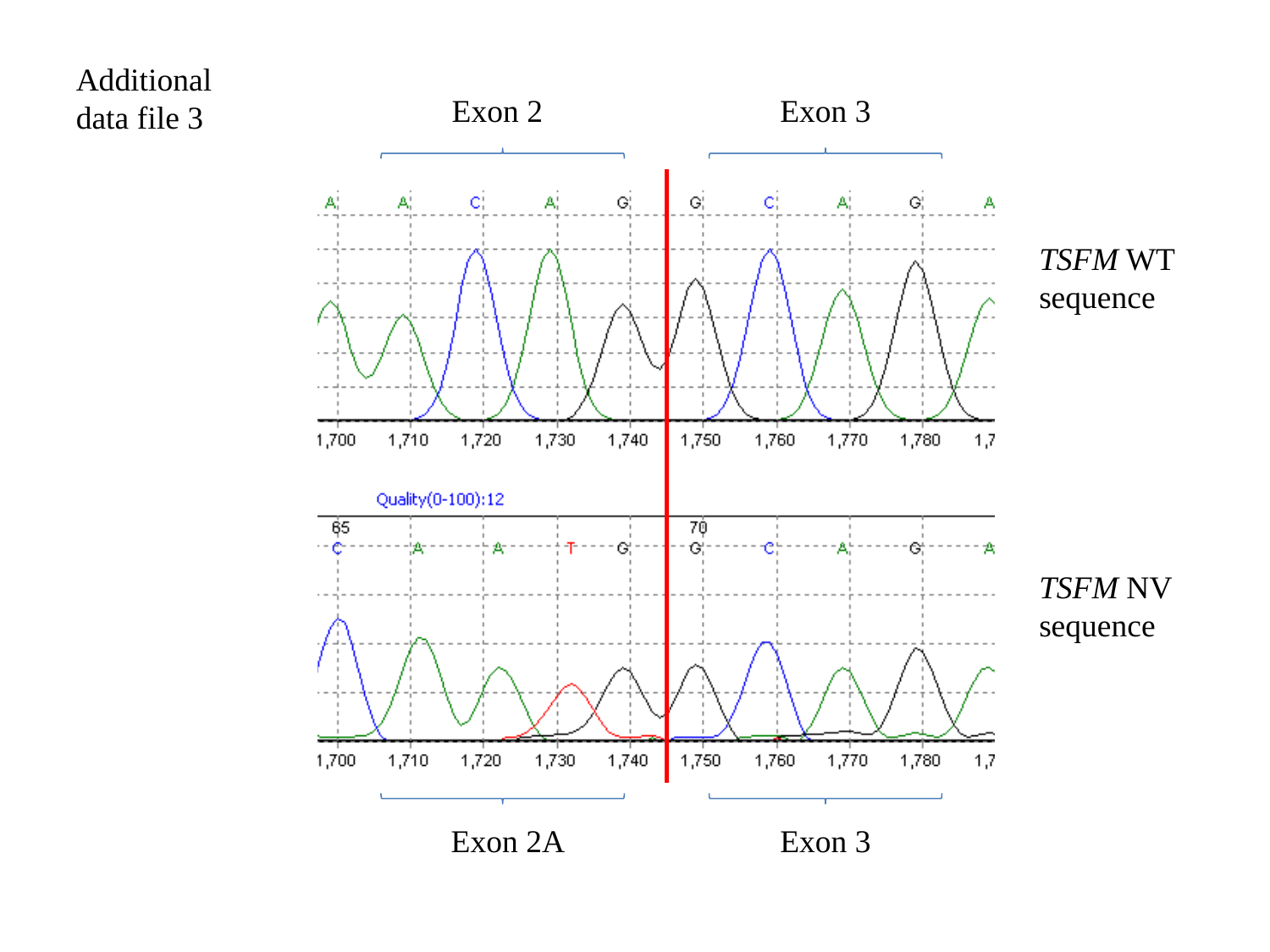

Additional data file 3
Exon 2
Exon 3
TSFM WT sequence
TSFM NV sequence
Exon 2A
Exon 3

Supplement: Additional file 3 — (Additional data file 3.ppt) is a figure showing the difference in nucleotide sequence between TSFM WT and TSFM NV transcript sequences. The title of the figure is “Electropherogram showing the changes to the TSFM transcript at the sequence level”. [file 1471-2164-14-627-S3.pptx]
