# Supplementary material for: The splice site variant rs11078928 may be associated with a genotype-dependent alteration in expression of GSDMB transcripts
Source: BMC Genomics. 2013 Sep 17;14:627. doi: 10.1186/1471-2164-14-627 (PMC3848490; doi:10.1186/1471-2164-14-627)
Supplement: Additional file 4 — (Additional data file 4.docx) is a table giving the age, BMI, white blood count, Platelet count and haemoglobin measurements for study participants. [file 1471-2164-14-627-S4.docx]

**Additional data file 4**. Cohort characteristics with reference to genotype, age, BMI, White cell count, platelet count and haemoglobin. BMI = Body Mass Index, Hb = haemoglobin, N/A = Not available.

| **GSDMB isoform specific expression** | **Median Age** | **Median BMI** | **Median white cell count** | **Median**  **Hb** | **Median Platelet count** |
| --- | --- | --- | --- | --- | --- |
| AA | 62 | 27 | 6 | 13.5 | 290 |
| AG | 61 | 25 | 6 | 13.9 | 247 |
| GG | 60 | 26 | 6 | 13.6 | 210 |
| P value (Kruskal Wallis test) | 0.987 | 0.611 | 0.740 | 0.618 | 0.923 |
| **GSDMB total expression** | **Median Age** | **Median BMI** | **Median white cell count** |  |  |
| AA | 60 | 25 | 5 | 14.5 | 206 |
| AG | 56 | 26 | 5 | 14.2 | 246 |
| GG | 54 | 25 | 5 | 13.0 | 222 |
| P value (Kruskal Wallis test) | 0.714 | 0.220 | 0.803 | 0.310 | 0.214 |
| TSFM isoform specific expression | **Median Age** | **Median BMI** | **Median white cell count** |  |  |
| CC | 46 | 25 | 5 | N/A | N/A |
| CT | 63 | 29 | 6 | N/A | N/A |
| TT | 37 | 22 | 4 | N/A | N/A |
| P value (Kruskal Wallis test) | *0.038* | 0.219 | 0.164 |  |  |
